# The complete mitochondrial genome of *Sporobolus alterniflorus* (loisel.) P.M. Peterson & Saarela (Poaceae) and phylogenetic analysis

**DOI:** 10.1080/23802359.2021.1907248

**Published:** 2021-03-31

**Authors:** Yanfeng Wang, Wancui Xie, Junhan Cao, Yingying He, Yang Zhao, Changfeng Qu, Jinlai Miao

**Affiliations:** a College of Marine Science and Biological Engineering, Qingdao University of Science and Technology, Qingdao, China; b Key Laboratory of Marine Eco-Environmental Science and Technology, First Institute of Oceanography, Ministry of Natural Resource, Qingdao, China; c Laboratory for Marine Drugs and Bioproducts, Qingdao Pilot National Laboratory for Marine Science and Technology, Qingdao, China

**Keywords:** *Sporobolus alterniflorus* (Loisel.) P.M. Peterson & Saarela, mitochondrial genome, phylogenetic analysis

## Abstract

The complete mitochondrial genome of *Sporobolus alterniflorus* was a circular molecule of 566,328 bp in length and encoded 64 genes, including 35 protein-coding genes, 24 tRNA genes, and 5 rRNA genes. The most common initiated codon was ATG and the most common termination codon was CAT. The overall A + T content was 55.96%. The phylogenomic analysis revealed that *Sporobolus alterniflorus* have a closest phylogenetic relationship with *Sorghum bicolor*.

*Sporobolus alterniflorus* was introduced to China to protect the coast since the 1960s (An et al. 2007). However, *S. alterniflorus* was identified as an invasive species (Li et al. 2009) because of its strong adaptability and reproductive ability. Looking for similar species for biological replacement has become a new method of *S*. *alterniflorus* control. In this study, we provided complete genome information of *S. alterniflorus* mitochondria for an in-depth study of similar species.

The wild samples of *S. alterniflorus* were gathered from the beach of Zhejiang Qinshan Nuclear Power Station (30°N, 121°E). The specimen was left with the Key Laboratory of Marine Bioactive Substances, the First Institute of Oceanography, Ministry of Natural Resource, China (Accession no. FIO2019301212). The mitogenome of *S. alterniflorus* was sequenced through Illumina NovaSeq PE150 at the Beijing Novogene Bioinformatics Technology Co., Ltd with the sequencing library of 320 bp. Then, the whole genome DNA was assembled by the SPAdes (Bankevich et al. 2012) and annotated by the DOGMA (Wyman et al. 2004).

The complete mitochondrial genome of *S. alterniflorus* (GenBank accession number: MT471321) was a circular molecule of 566,328 bp in length with base compositions of 27.96% A, 21.89% C, 22.15% G, and 27.99% T, exhibiting AT bias (55.96%). The mitogenome encoded 64 genes, including 35 protein-coding genes (PCGs), 24 tRNA genes, 5 rRNA genes. The 35 PCGs included NADH dehydrogenase subunit, cytochrome c oxidase subunit, ATPase subunits, ribosomal proteins, cytochrome c biogenesis, maturase and membrane transporter, apocytochrome b. The most common initiated codon was typical ATG and the most termination codon was CAT. The 24 tRNA genes (tRNA-Pro, tRNA-Ser, tRNA-Met, tRNA-Asn, tRNA-Asp, tRNA-His, tRNA-Arg, tRNA-Cys, tRNA-Glu, tRNA-Gln, tRNA-Tyr, tRNA-Phe, tRNA-Trp, tRNA-Lys), which sized from 70 bp (trnR) to 91 bp (trnS). The lengths of rRNA were 120 bp (rrn5), 1969 bp (rrnS), 3547 bp (rrnL).

To investigate the evolutionary relationship of *S. alterniflorus*, we performed the phylogenetic analysis based on nucleotide sequences of 15 complete mitochondrial genomes. The sequences were aligned by the muscle method. Poisson correction model were used to calculate the genetic distances. A maximum-likelihood phylogenetic tree (Figure 1) was implemented with 3000 bootstrap replications. All the cladistics analyses were performed by Mega (v7.0.26) (Sudhir et al. 2016). Our results support that *S. alterniflorus* have a closest phylogenetic relationship with *Sorghum bicolor*. The *S. alterniflorus* was placed in the subfamily Chloridoideae by Soreng et al. (2017). However, our result does not support this classification since the complete mitogenome of the type genus of the subfamily is not available. The complete mitochondrial genome of *S. alterniflorus* will provide useful genetic information to study the genetic evolution of Poaceae.

**Figure 1.**
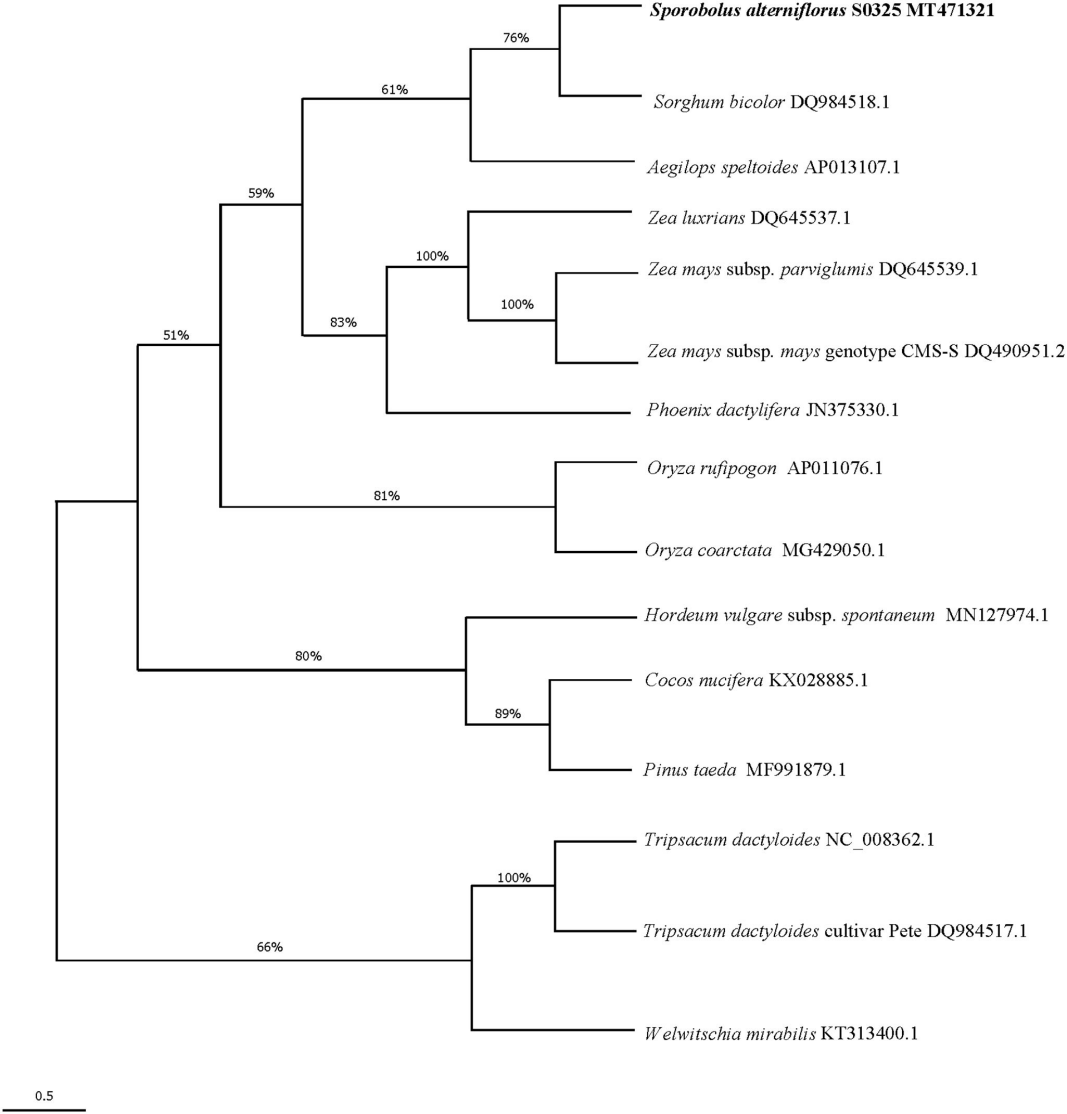
Maximum likelihood phylogenetic tree based on 15 complete mitochondrial genome.

## Data Availability

The genome sequence data that support the findings of this study are openly available in GenBank of NCBI at (https://www.ncbi.nlm.nih.gov/) under the accession no. MT471321 The associated BioProject, SRA, and Bio-Sample numbers are PRJN A691393, SRR13512336, and SAMN17292058, respectively.
